# Pomological and Biochemical Properties of Blackberry (*Rubus fruticocus*) Genotypes

**DOI:** 10.1002/fsn3.70591

**Published:** 2025-07-14

**Authors:** Hande Dogan, Erdal Aglar, Burhan Ozturk, Onur Tekin, Davut Alan, Ahmet Sumbul

**Affiliations:** ^1^ Department of Horticulture, Faculty of Agriculture Van Yuzuncu Yil University Van Turkey; ^2^ Faculty of Arts, Design and Architecture, Department of Landscape Architecture Munzur University Tunceli Turkey; ^3^ Department of Horticulture Faculty of Agriculture, Ordu University Ordu Turkey; ^4^ Suşehri Timur Karabal Vocational School, Department of Plant and Animal Production Sivas Cumhuriyet University Sivas Turkey

**Keywords:** antioxidant activity, organic acids, phenolic compounds, total flavonoid, total monomeric anthocyanin

## Abstract

The aim of this study was to examine the genotypes of wild blackberries growing naturally in Tunceli province in terms of fruit characteristics and to determine the ones with superior characteristics. Nine different genotypes were determined by population screening and 100 fruits were collected from each genotype. The physical properties such as fruit weight, length, width, as well as color, chemical content, and antioxidant properties were examined. Statistically significant differences were found among the genotypes; fruit weight, length, and width varied between 0.71 and 1.19 g, 10.08 and 12.63 mm, and 12.25 and 14.33 mm, respectively. The G9 genotype had the largest fruits, and the G6 genotype had the smallest fruits. In fruit color analyses, significant differences were observed among the genotypes, and the G2 genotype stood out with the highest L* value (20.05), but G9 had the lowest a* value (0.92). Total soluble solids (TSS) content varied between 11.95% in G2 and 21.10% in G7. Vitamin C content was highest in the G6 genotype (54.46 mg 100 g^−1^), while G9 had the lowest vitamin C content (22.66 mg 100 g^−1^). Significant differences were also observed among the genotypes in terms of phenolic content, flavonoids, anthocyanins, and antioxidant activity. As a result, in this study, significant differences in organic acid contents were determined among wild blackberry genotypes in Tunceli province. The G1 genotype was the richest in malic acid, while the G6 was the genotype with the highest ascorbic acid level. Various differences were also found among the genotypes in terms of oxalic acid, citric acid, and other organic acid contents. These findings may constitute an important resource in determining the commercial production and potential health benefits of wild blackberries. In the selection of blackberry genotypes, consideration of these organic acid contents is important, especially in terms of taste and nutritional value.

## Graphical abstract

In this study, blackberry genotypes naturally grown in Tunceli were examined, and those with superior characteristics were identified. The results showed significant differences among the genotypes in terms of fruit weight, length, width, color values, total soluble solids (TSS), titratable acidity, vitamin C, antioxidant activity, organic acids, and individual phenolic compounds. While the G9 genotype had the largest fruits, the G6 genotype stood out with its high phenolic, flavonoid, and anthocyanin content. The G2 genotype was distinguished by its high color values and acidity content. The levels of phenolic compounds in different blackberry genotypes show great variation; for example, G1, G2, and G6 genotypes have high levels of phenolic compounds, while G4 and G5 genotypes have low levels. In particular, G2 and G1 are rich in hydroxybenzoic acid, caffeic acid, and ferulic acid, while some genotypes such as G4 and G5 have low levels of phenolic compounds. The G7 genotype has the highest oxalic acid level, while G8 has the lowest level. In terms of other acids, G1 is the richest genotype in malic acid, G6 has the highest value in ascorbic acid, and citric acid is at the lowest level in G6. As a result of the study, the G9 genotype had the largest fruits, while the G6 genotype stood out with its high phenolic, flavonoid, and anthocyanin content; also, G2 attracted attention with its high color values and acidity content, and G1 with its richness in malic acid.

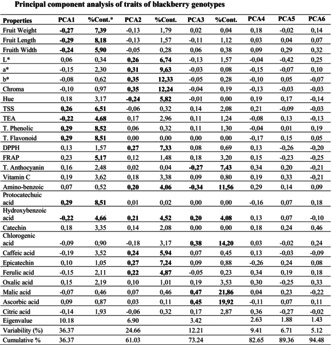

**Abbreviations**
GAE: gallic acid equivalent
pH: power of hydrogen
QE: quercetin equivalent
TE: trolox equivalent
TEA: titratable acidity
TMA: total monomeric anthocyanin
TSS: total soluble solids content

## 1 Introduction

Anatolia is one of the homelands of many fruit species worldwide and is considered a globally important region in terms of plant population diversity. These lands also stand out with the rich biological diversity that Türkiye has, and numerous wild fruit species grow naturally in nature (Artik and Eksi 1988). Blackberry (*Rubus fructicosus*) is a member of the Rubus genus of the Rosaceae family and is classified into two subgenera, *Rubus Ideaobatus Focke* and *Rubus Euabatus Focke* (Baltacioglu and Velioglu 2003; Galletta et al. 1998). Wild blackberry has been collected and used in human nutrition since ancient times, and this plant, which has a history of 8000 years, is also known for its healing properties. Since Türkiye has a region that is the homeland of blackberries, this fruit grows naturally in different regions of our country (Toker 2017). Therefore, Türkiye has a very large wild blackberry population and forms an important part of biodiversity. The great potential of blackberries in terms of health makes them very valuable. Blackberries are also frequently used for nutraceutical purposes due to their high flavonoid content, strong antioxidant capacity, and contribution to the prevention of cardiovascular diseases. In addition, the antioxidant compounds they contain play an important role in the fight against free radicals and strengthen the body's immune system. The health benefits of these biologically active compounds have become the focus of many studies in recent years (Basu et al. 2012). The rich content of blackberries, especially in flavonoids, anthocyanins, and polyphenols, provides them with high antioxidant capacity, which makes them suitable for use in many areas such as fresh consumption, processed food, and the pharmaceutical industry. This potential of blackberries varies greatly depending on the ecological conditions in which they grow. Significant differences in fruit quality traits and biologically active compound contents are observed among blackberries grown in different growing areas (such as various regions of Türkiye, Italy, and Mexico). For example, it has been reported that blackberries exhibit significant variations in taste, color, acidic properties, and antioxidant activity in regions of Türkiye with different climate and soil characteristics (Toker 2017). Such variations are very important to better understand the effects of local climatic conditions on the chemical content of blackberries. In this context, the differences that blackberries show depending on the environmental factors in which they grow are considered as an indicator of genetic diversity and adaptability. However, research on blackberries in Türkiye has generally focused on the adaptation of alien, thornless species, and there are significant gaps in this regard. Genetic diversity and selection studies of wild blackberry populations have not yet been addressed comprehensively enough. In most regions, especially in microclimate zones such as Tunceli, scientific studies on wild blackberries are limited. Tunceli has numerous microclimate zones with its mountainous structure and river systems, which create important ecological opportunities for wild blackberry populations in the region. Blackberries in Tunceli are likely to exhibit different biological characteristics due to the unique ecological conditions in the region. In this context, the aim of this study is to examine the genotypes of wild blackberries growing naturally in Tunceli province in terms of fruit characteristics and to determine those with superior characteristics based on selection criteria. This study aims to provide more information about wild blackberry populations in Türkiye and to help select more productive and healthy genotypes by evaluating the genetic diversity of these populations.

## 2 Materials and Methods

The study was carried out in Karşılar Village of Tunceli Central District, which has a significant natural wild rosehip potential and is located in the Upper Euphrates Basin of the Eastern Anatolia Region, at 39°10′30.79″ North latitude and 39°26′28.45″ East longitude. The research area is under the influence of a continental climate, with hot and dry summers, long, cold, and snowy winters; the annual average temperature is 12.5°C, and annual precipitation varies between 550 and 1080 mm. A total of nine genotypes were identified through a population screening, and 100 fruits were collected from each genotype during harvest. Generally, ripe blackberry fruits are black, and the fruit stalks are slightly brown. The harvest of blackberry fruits was carried out when the fruits were easily separated from the cluster. The study was carried out in three replications, with 30 fruits in each replication. The following measurements and analyses were performed on the fruits.

### 2.1 Fruit Weight (g), Width and Length (mm)

Thirty fruits were randomly selected and weighed on a precision balance with 0.01 g accuracy. The average fruit weight was calculated by dividing the total weight by the number of fruits. The widest point at the equatorial part of the fruit was measured as the fruit width, and the distance between the stem end and the opposite end was measured as the fruit length. These measurements were taken on 20 fruit samples using a digital caliper with 0.01 mm accuracy.

### 2.2 Fruit Color

Twenty fruits were randomly selected among the harvested samples. The external color of the fruits was measured using a “CR400 model Minolta Colorimeter,” and L*, a*, and b* values were determined by calibrating the device against a white plate (Cemeroglu 1982).

### 2.3 Total Soluble Solids (%)

A sufficient amount of fruit was juiced using an electric juicer and filtered through cheesecloth. The juice was analyzed for total soluble solids (TSS) content using a digital refractometer (PAL‐1, Atago, USA), and the values were expressed as a percentage.

### 2.4 Titratable Acidity (%)

A 10 mL sample of juice, obtained for TSS determination, was diluted with 10 mL distilled water and titrated with 0.1 mol L^−1^ sodium hydroxide (NaOH) until reaching pH 8.1. The amount of NaOH used was expressed as citric acid equivalent (g citric acid per 100 mL juice).

### 2.5 Vitamin C

Vitamin C was measured using a Reflectoquant plus 10 device (Merck RQflex plus 10, Türkiye). A 0.5 mL juice sample was taken and diluted to 5 mL with 0.5% oxalic acid. An ascorbic acid test kit (Catalog no: 116981, Merck, Germany) was briefly dipped in the solution for 2 s and allowed to oxidize outside the solution for 8 s, then read in the Reflectoquant device within 15 s. Results were expressed in mg 100 g^−1^ (Ozturk et al. 2019).

### 2.6 Total Phenolic Compounds

Total phenolics were determined using the Folin–Ciocalteu reagent, as described by Ozturk et al. (2019). Initially, 600 μL of fruit extract was mixed with 4.0 mL distilled water, followed by the addition of 100 μL Folin–Ciocalteu reagent and 300 μL of 2% sodium carbonate solution. After a 2‐h incubation at room temperature, the solution's absorbance was read at 760 nm using a UV–vis spectrophotometer (Shimadzu, Japan). Results were expressed as mg gallic acid equivalents (GAEs) 100 g^−1^ on fresh weight.

### 2.7 Total Flavonoids Content

Total flavonoids were measured using a modified version of the method by Zhinsen et al. (1999). A 600 μL extract was mixed with 3.7 mL methanol, then 100 μL of 10% aluminum nitrate [Al(NO₃)₃] and 0.1 M ammonium acetate were added, reaching a final volume of 4.5 mL. After a 40‐min incubation in darkness, the absorbance was read at 415 nm with a UV–vis spectrophotometer, and results were expressed as mg quercetin equivalents (QEs) 100 g^−1^ on fresh weight.

### 2.8 Antioxidant Activity

The DPPH radical scavenging activity was determined using a modified method from Blois (1958). A 500 μL extract was mixed with 2.5 mL ethanol to a final volume of 3.0 mL. DPPH radical solution (0.1 mM in ethanol) was added to reach a final volume of 4 mL, mixed with a vortex, and incubated in darkness for 30 min. Absorbance was measured at 517 nm, with results expressed as mmol Trolox equivalents (TEs) 100 g^−1^ on fresh fruit. The FRAP (Ferric Reducing Antioxidant Power) assay was conducted as described by Ozturk et al. (2019). A 100 μL fruit extract was combined with phosphate buffer (1.15 mL, 0.2 M, pH 6.7) and potassium ferricyanide (1.25 mL, 1%). After a 20‐min incubation at 50°C, the mixture was cooled, and trichloroacetic acid (1.25 mL, 10%) and ferric chloride (0.25 mL, 0.1%) were added. Absorbance was read at 700 nm, and results were expressed as mmol TE 100 g^−1^ on fresh fruit.

### 2.9 Total Monomeric Anthocyanin

Total monomeric anthocyanin (TMA) was measured using the pH differential method by Giusti et al. (1999). Extracts were prepared in pH 1.0 and 4.5 buffers, and absorbance was measured at 533 nm and 700 nm. Total anthocyanin content was calculated as cyanidin‐3‐glucoside equivalents, expressed as μg cyn‐3‐gluc g^−1^ on fresh weight.

### 2.10 Individual Phenolic Compounds

Individual phenolic compounds were determined using the method by Rodrıguez‐Delgado et al. (2001). In this method, 5 g of blackberry fruit samples were mixed with distilled water in a 1:1 ratio and centrifuged at 15,000 rpm for 15 min. The supernatants were filtered through a coarse filter, followed by a 0.45 μm membrane filter (Millipore Millex‐HV Hydrophilic PVDF, Millipore, USA), and then injected into the HPLC (Agilent HPLC 1100 series G 1322 A, Germany). Chromatographic separation was performed using a DAD detector and a 250 × 4.6 mm, 4 μm ODS column (HiChrom, USA). Solvent A, methanol: acetic acid: water (10:2:28), and Solvent B, methanol: acetic acid: water (90:2:8), were used as mobile phases (Merck, 99% purity). Spectral measurements were carried out at 254 and 280 nm, and the flow rate and injection volume were set to 1 mL min^−1^ and 20 μL, respectively. The results were expressed as mg 100 g^−1^ on fresh weight.

### 2.11 Organic Acids

A measure of 5 g of fruit sample was homogenized with 0.009 N H₂SO₄ in a 1:1 ratio and mixed on a shaker for 1 h. The samples were centrifuged at 15,000 rpm for 15 min. The supernatant was filtered through 0.45 μm hydrophilic PTFE membrane filter discs and then through a SEP‐PAK C18 cartridge for HPLC analysis. Organic acids were analyzed using the method developed by Bevilacqua and Califano (1989) on an HPLC (Agilent HPLC 1100 series G 1322 A, Germany) equipped with an Aminex HPX‐87H, 300 x 7.8 mm column (Bio‐Rad Laboratories, Richmond, CA, USA). The results were expressed as mg 100 g^−1^ on fresh weight.

### 2.12 Statistical Analyses

Data were analyzed using a randomized plot design with SAS Software (SAS Version V.8, SAS Institute, Cary, N.C., SAS, 2005). Differences among means were determined using Tukey's test. Correlation analysis, which reveals the relationship between examined traits, and Heatmap and PCA analysis, which explain the relationship between genotypes and examined traits, were performed using the JMP Pro 17 statistical package program.

## 3 Results and Discussion

### 3.1 Fruit Size

The data on fruit size is presented in Table 1. Examination of the table reveals statistically significant differences in terms of fruit weight, length, and width. Fruit weight, length, and width values ranged from 0.71 to 1.19 g, 10.08 to 12.63 mm, and 12.25 to 14.33 mm, respectively. The largest fruits were harvested from the G9 genotype, while G6 had the smallest fruits. Compared with similar studies, the fruits were found to be smaller than those of cultivated varieties but comparable to wild species (Table 1). Fruit size is an important criterion for both fresh consumption and processing, with a desired fruit weight of 8–10 g in blackberries (Clark and Finn 2011). Fruit size can vary depending on the variety, ecological factors of the growing region, and the cultivation status of the species, with wild blackberry fruits generally being smaller. In a study by Yilmaz et al. (2009), fruit weight in blackberry varieties ranged from 1.2 to 5.4 g, whereas wild genotypes had an average fruit weight between 0.4 and 1.2 g. In Türkiye, wild blackberry fruit weight varies between 1.50 and 2.11 g (Celik et al. 2003), while cultivated varieties range between 2.00 and 6.82 g (Gercekcioglu 1999; Cangi and Islam 2003; Agaoglu et al. 2007). In a study conducted by Aglar et al. (2021) on wild blackberry genotypes naturally growing in the Kelkit Valley, the average fruit width, length, height, and weight were found to be 15.84, 15.37, 15.72 mm, and 2.16 g, respectively. In another study, Mikulic‐Petkovsek et al. (2012) reported the fruit weight of blackberry cultivars Bestma, Cacanska, Smoothstem, Navaho, Thornfee, and Loch Ness as ranging from 5.87 to 8.38 g, indicating that ecological factors and genotype might contribute to different results in similar studies.

**TABLE 1:** Fruit weight, fruit length, and fruit width in blackberry genotypes naturally grown in Tunceli.

Genotypes	Fruit weight (g)	Fruit length (mm)	Fruit width (mm)
G1	0.99ab	11.34abc	13.32ab
G2	0.94bc	11.55abc	13.50ab
G3	0.91bc	10.92bc	12.77b
G4	0.95b	11.71ab	12.85b
G5	0.94bc	11.12abc	12.64b
G6	0.71c	10.08c	12.47b
G7	0.81bc	10.59bc	12.25b
G8	0.85bc	11.36abc	13.13ab
G9	1.19a	12.63a	14.33a

*Note:* Means in columns with the same letter do not differ according to Tukey's test at *p* < 0.05.

### 3.2 Fruit Color

The color values (L*, a*, b*, chroma, and hue°) of the blackberries used in the study are presented in Table 2. Significant variations in color values were observed among genotypes. The highest L* value (20.05) was recorded in genotype G2, while the lowest (18.21) was also recorded in G2. In fruit color analysis, +a* represents red and −a* green. The lowest a* value (0.92) was recorded in G9, while the highest (4.20) was recorded in G2. The +b* value indicates yellowness, while −b* suggests blueness. In this study, b* values ranged from 2.57 to 4.02, with the lowest in G9 and the highest in G2. Chroma, which indicates the vividness or dullness of fruit color, varied significantly between genotypes, ranging from 2.61 (G9) to 5.93 (G2). As the Hue° value decreases, fruit color shifts toward red. The lowest hue° value (47.41) was recorded in G2, and the highest (72.28) in G7 (Table 2). When compared with previous studies, it is observed that the L value is at similar levels, while chroma and hue values are significantly lower. Mikulic‐Petkovsek et al. (2012) reported L, chroma, and hue values of blackberry cultivars Bestma, Cacanska, Smoothstem, Navaho, Thornfee, and Loch Ness as 21.80–23.1, 24.03–24.64, and 347.88–348.65, respectively.

**TABLE 2:** Fruit color in blackberry genotypes naturally grown in Tunceli.

Genotypes	L*	a*	b*	Chroma	Hue angle
G1	19.13abc	0.97b	2.93b	3.09b	72.01a
G2	20.05a	4.20a	4.02a	5.93a	47.41c
G3	18.69bc	1.03b	2.79b	2.98b	69.89a
G4	18.22c	1.14b	2.86b	3.08b	68.39ab
G5	19.25abc	1.71b	2.77b	3.28b	59.37b
G6	18.81abc	1.64b	2.98b	3.48b	64.26ab
G7	19.87ab	0.92b	2.89b	3.06b	72.28a
G8	18.46c	1.08b	3.02b	3.22b	71.67a
G9	18.21c	1.41b	2.57b	2.61b	63.23ab

*Note:* Means in columns with the same letter do not differ according to Tukey's test at *p* < 0.05.

### 3.3 TSS Content, Titratable Acidity, and Vitamin C

As shown in Table 3, there were statistically significant differences among blackberry genotypes regarding TSS, titratable acidity, and vitamin C. The TSS varied between 11.95% (G2) and 21.10% (G7). The highest titratable acidity (2.32%) was measured in G2, while G3 showed the lowest acidity. The vitamin C content ranged from 22.66 mg 100 g^−1^ (G9) to 54.46 mg 100 g^−1^ (G6) (Table 3). The chemical composition of blackberries can vary depending on species, variety, growing conditions, harvest season, and timing. The TSS content, an important parameter influencing consumer preferences in the food industry Abiodun and Akinoso (2014) varies between 4% and 12% in blackberries. Cultivation conditions can affect TSS, and wild species generally have higher TSS than cultivated varieties (Yilmaz et al. 2009). When compared with similar studies, TSS levels in the genotypes studied here appear relatively high. In a study in Colombia, TSS among 10 blackberry genotypes ranged from 5.68% to 7.55% (Sánchez‐Betancourt et al. 2020). Eskimez et al. (2019) reported TSS levels between 7.1% and 9.5% in blackberries, while Moraes et al. (2020) noted variations in TSS across varieties Guarani, Tupy, Xavante, and BRS Xingu, with values of 7.5%, 7.3%, 9.0%, and 9.0%, respectively. In Türkiye, soluble solids in cultivated blackberries range from 8.98% to 20.2%, while acidity levels vary between 1.0% and 3.1%, with TSS in wild blackberries ranging from 11.3% to 13.1% and acidity from 0.7% to 1.0% (Gercekcioglu 1999; Agaoglu et al. 2007). In a study on wild blackberries from Kelkit Valley, Aglar et al. (2021) reported an average TSS of 9.2%. Mikulic‐Petkovsek et al. (2012) found TSS values between 8.42% and 12.21% in blackberry varieties Bestma, Cacanska, Smoothstem, Navaho, Thornfee, and Loch Ness.

**TABLE 3:** Total soluble solids, titratable acidity, and vitamin C in blackberry genotypes naturally grown in Tunceli.

Genotype	TSS (%)	TA (%)	Vitamin C (mg 100 g^−1^)
G1	18.80d	2.24b	50.46b
G2	11.95i	2.32a	39.55d
G3	19.20c	0.96i	51.06d
G4	13.75 h	1.33f	32.50c
G5	17.55e	1.44e	24.10f
G6	19.85b	1.17 g	54.46a
G7	21.10a	1.12 h	42.13c
G8	16.35f	1.77c	41.13 cd
G9	14.75 g	1.67d	22.66f

*Note:* Means in columns with the same letter do not differ according to Tukey's test at *p* < 0.05.

### 3.4 Total Phenolics, Total Flavonoids, Total Monomeric Anthocyanins, and Antioxidant Activity

As shown in Table 4, the total phenolic content, which varied depending on the genotype, was found to range from 459.20 mg GAE 100 g^−1^ (G9) to 1081.70 mg GAE 100 g^−1^ (G6). The total flavonoid and TMA values ranged from 158.40 mg QE 100 g^−1^ (G9) to 868.13 mg QE 100 g^−1^ (G6) and from 76.96 μg cyn‐3‐gluc g^−1^ (G9) to 234.59 μg cyn‐3‐gluc g^−1^ (G3), respectively (Table 4). Significant differences in antioxidant activity were observed among genotypes, with FRAP and DPPH values, which are used to determine antioxidant activity, ranging from 27.73 mmol TE 100 g^−1^ (G9) to 61.16 mmol TE 100 g^−1^ (G6) and from 6.12 mmol TE 100 g^−1^ (G9) to 7.55 mmol TE 100 g^−1^ (G2), respectively (Table 4). Blackberries are rich in phenolic compounds such as anthocyanins, flavonols, chlorogenic acid, and proanthocyanidins, which have high biological activity and provide antioxidant health benefits (Cho et al. 2005; Koca and Karadeniz 2009; Zia‐Ul‐Haq et al. 2014). The content and concentration of bioactive compounds in blackberries vary depending on species, variety, ecological conditions, and cultural practices. Wild blackberries are richer in terms of total phenolics (Reyes‐Carmona et al. 2005; Yilmaz et al. 2009; Mikulic‐Petkovsek et al. 2012) and antioxidants (Yildiz et al. 2010) compared to cultivated varieties. Yilmaz et al. (2009) suggested that increased exposure of wild fruit species to extreme temperatures and their susceptibility to pests and diseases may promote the synthesis of antioxidant enzymes as a defense mechanism, leading to an increase in polyphenolic concentration. Yildiz et al. (2010) reported that wild genotypes had an average total phenolic content of 381 mg GAE 100 g^−1^ FW, higher than that of cv. Chester (310 mg 100 g^−1^ FW). In a study conducted to determine the physicochemical properties of wild and cultivated blackberry varieties (Yilmaz et al. 2009), significant differences were found between varieties and wild genotypes in terms of physicochemical characteristics, with the total phenolic content of cultivated varieties ranging from 584 to 788 mg 100 g^−1^ FW and wild genotypes from 610 to 1455 mg 100 g^−1^ FW. The same study found that wild genotypes had lower antioxidant activity, with the average antioxidant activities of wild genotypes and cultivated varieties being 76.2% and 81.9%, respectively.

**TABLE 4:** Total phenolics, total flavonoid, total monomeric anthocyanin, and antioxidant activity (DPPH and FRAP) in blackberry genotypes naturally grown in Tunceli.

Genotypes	Total phenolics (mg GAE 100 g^−1^)	Total flavonoid (mg QE 100 g^−1^)	Total monomeric anthocyanin (μg cyn‐3‐gluc g^−1^)	DPPH FRAP (mmol TE 100 g^−1^)	
G1	623.78bc	255.41ef	114.69d	7.49a	58.17a
G2	523.09 cd	212.43f	108.48d	7.55a	43.02b
G3	667.98bc	428.54d	234.59a	7.40a	56.02a
G4	569.75b‐d	423.63d	133.06c	6.38b	41.55b
G5	564.84b‐d	297.16e	77.20e	7.42a	54.27a
G6	1081.78a	868.13a	120.40 cd	7.44a	61.16a
G7	1065.80a	685.17b	150.19b	7.41a	60.61a
G8	685.20b	604.13c	152.92b	6.66b	39.71b
G9	459.20d	158.40 g	76.95e	6.12b	27.73c

*Note:* Means in columns with the same letter do not differ according to Tukey's test at *p* < 0.05.

### 3.5 Individual Phenolic Compounds

The study found that aminobenzoic acid levels were highest in G3 (4.65 mg 100 g^−1^) and G2 (4.31 mg 100 g^−1^) genotypes and relatively lower in G5 (2.68 mg 100 g^−1^) and G4 (2.95 mg 100 g^−1^) genotypes (Table 5). Known for its antioxidant properties, aminobenzoic acid is widely found in various fruits including blackberries. Acosta‐Montoya et al. (2010) discussed the antioxidant potential of blackberry phenolic compounds and emphasized the role of these compounds in neutralizing free radicals. High aminobenzoic acid levels in genotypes such as G3 and G2 suggest that these genotypes may have superior antioxidant capacities (Table 5). These findings are consistent with the findings of Kaume et al. (2012) who observed high antioxidant activity in blackberry cultivars. For protocatechin, G6 (3.40 mg 100 g^−1^) showed the highest content, while G1 (0.43 mg 100 g^−1^) had the lowest level (Table 5). Protocatechin is a flavonoid with strong antioxidant activity (Lee et al. 2012). This supports that G6 could potentially be a strong antioxidant source. On the other hand, low levels in genotypes such as G1 suggest that they may exhibit less antioxidant activity, although this may vary depending on different factors such as the presence of other phenolic compounds. Hydroxybenzoic acid levels were significantly higher in G2 (14.53 mg 100 g^−1^) and G1 (13.86 mg 100 g^−1^) (Table 5), which is consistent with the findings of Gil‐Martínez et al. (2023) on the antioxidant, anti‐inflammatory, and procancer properties of hydroxybenzoic acids. These compounds are critical for fruit health, especially in terms of reducing oxidative stress, and have been supported by studies such as Castañeda‐Ovando et al. (2009). The significant presence of hydroxybenzoic acids in these genotypes suggests potential health benefits in terms of reducing oxidative damage and inflammation. In terms of catechin, G6 (0.11 mg 100 g^−1^) and G7 (0.10 mg 100 g^−1^) showed the highest production, while a few genotypes such as G4 (0.00 mg 100 g^−1^) and G5 (0.00 mg 100 g^−1^) did not have detectable levels (Table 5). Catechin is a flavonoid with well‐documented health benefits such as cardiovascular protection, supporting the antioxidant theory proposed by Sariburun et al. (2010). The effect of catechin in reducing cardiovascular risks in other fruit studies (Moreno‐Medina et al. 2023) further highlights the potential health benefits of these genotypes. In terms of chlorogenic acid, G9 (7.80 mg 100 g^−1^) and G1 (5.73 mg 100 g^−1^) exhibited the highest levels, while G8 (1.20 mg 100 g^−1^) and G2 (1.92 mg 100 g^−1^) were at relatively lower levels (Table 5). Chlorogenic acid is not only a potent antioxidant but also exhibits antiobesity effects, an issue addressed in the studies of Boccellino and D'Angelo (2020). High levels of G9 and G1 suggest that these genotypes may contribute to metabolic health benefits, particularly regarding weight management and insulin sensitivity, findings consistent with work by Gowd et al. (2018). The high levels of caffeic acid in G2 (11.64 mg 100 g^−1^) and G1 (7.43 mg 100 g^−1^) are noteworthy (Table 5), as caffeic acid is known for its antioxidant, anti‐inflammatory, and antimicrobial properties (Toshima et al. 2021). The low levels in G7 (1.78 mg 100 g^−1^) and G8 (0.71 mg 100 g^−1^) may indicate that these genotypes may be less beneficial in these aspects (Table 5), which is in line with the general understanding of the role of caffeic acid in health and prodisease (Qian et al. 2017). Epicatechin, one of the flavonoids associated with cardiovascular health, was found in significant amounts in G6 (2.46 mg 100 g^−1^) and G2 (2.07 mg 100 g^−1^) (Table 5). This compound is found in high levels in berries, especially blackberries, which have been associated with reduced heart disease risks and have a strong antioxidant profile (Turan et al. 2023). The high epicatechin levels in these genotypes further highlight their potential to exert positive effects on cardiovascular health. Finally, ferulic acid levels were high in G2 (7.32 mg 100 g^−1^) and G1 (4.95 mg 100 g^−1^) (Table 5), consistent with previous studies reporting strong antioxidant and antiaging properties of ferulic acid (Blando et al. 2018). Lower levels in genotypes such as G5 (1.16 mg 100 g^−1^) and G4 (2.27 mg 100 g^−1^) suggest that these genotypes may be less beneficial in combating oxidative stress. In conclusion, the phenolic profiles of different blackberry genotypes differ significantly. Some genotypes, such as G1, G6, and G7, may have superior antioxidant and health‐supporting properties, showing high levels of phenolic compounds such as hydroxybenzoic acid, caffeic acid, and ferulic acid. These findings are in line with existing research emphasizing the role of phenolic compounds in health and combating oxidative stress and inflammation, and support the potential of certain genotypes in functional foods and dietary supplements.

**TABLE 5:** Individual phenolic compounds (mg 100 g^−1^) in blackberry genotypes naturally grown in Tunceli.

Genotypes	Aminobenzoic	Protocatechin	Hydroxybenzoic	Catechin
G1	3.10ef	0.43h	13.86b	0.04c
G2	4.31b	0.62g	14.53a	0.06abc
G3	4.65a	1.52d	4.08e	0.08abc
G4	2.95g	1.03f	5.79d	0.00d
G5	2.68h	1.14e	3.44f	0.00d
G6	3.21e	3.40a	3.21g	0.11a
G7	3.98c	2.60b	0.93h	0.10 ab
G8	3.77d	1.86c	0.85h	0.05bc
G9	3.03fg	0.56g	6.92c	0.05bc

Genotypes	Chlorogenic acid	Caffeic acid	Epicatechin	Ferulic acid
G1	5.73b	7.43b	1.12d	4.95c
G2	1.92g	11.64a	2.07b	7.32a
G3	2.87f	6.36c	0.00f	5.81b
G4	4.46e	6.04d	0.00f	2.27f
G5	4.99d	4.50f	0.18e	1.16h
G6	5.30c	3.96g	2.46a	2.49e
G7	2.92f	1.78h	1.05d	2.77d
G8	1.20h	0.71ı	1.57c	2.00g
G9	7.80a	4.76e	0.00f	4.92c

*Note:* Means in columns with the same letter do not differ according to Tukey's test at *P* < 0.05.

### 3.6 Organic Acids

Organic acids are critical components that determine the quality of fruits like blackberry, influencing their taste profile, pH balance, and nutritional value. Rodríguez et al. (2021) indicated that the organic acids in blackberry, particularly by contributing to the acidic taste profile, directly affect fruit quality. Organic acid contents in different blackberry genotypes naturally grown in Tunceli are shown in Table 6. Oxalic Acid: While the G7 genotype had the highest oxalic acid content with a value of 0.33 mg 100 g^−1^, this value was recorded at the lowest level with 0.18 mg 100 g^−1^ in the G8 genotype. The oxalic acid amounts of G1, G3, and G6 among the other genotypes ranged from 0.23–0.26 mg 100 g^−1^. Malic Acid: The genotype containing the highest malic acid was G1 with 15.68 mg 100 g^−1^, followed by G6 with 11.00 mg 100 g^−1^. While the malic acid level was found at a moderate level with 8.48 mg 100 g^−1^ in the G2 and G9 genotypes, G3 (5.86 mg 100 g^−1^) and G7 (5.95 mg 100 g^−1^) had relatively lower values. Ascorbic Acid: G6 showed the highest value in terms of ascorbic acid with 1.04 mg 100 g^−1^. While G2 (0.65 mg 100 g^−1^) and G9 (0.67 mg 100 g^−1^) are at medium levels, G3 (0.46 mg 100 g^−1^) and G8 (0.52 mg 100 g^−1^) contain more limited amounts of ascorbic acid. The G1 genotype can be evaluated at a medium‐high level in terms of this acid type with 0.89 mg 100 g^−1^. Citric Acid: While G1 (0.16 mg 100 g^−1^) and G4 (0.11 mg 100 g^−1^) are among the prominent genotypes in terms of citric acid, G6 (0.06 mg 100 g^−1^) has the lowest value. In the G2, G5, G7, and G9 genotypes, the amount of citric acid was observed at moderate levels. In terms of malic acid content, G1 was found at much higher levels than other genotypes, which showed that it may be important in terms of sweetness and acidic properties. The genotype with the highest level of ascorbic acid was G6, while G1 was at intermediate levels. The findings of this study reveal that blackberry genotypes in Tunceli show significant differences in terms of organic acid contents (Table 6). In particular, malic acid stands out as the main organic acid of blackberries, which is consistent with the studies conducted by Kafkas et al. (2006). This result, where malic acid is dominant but citric acid is absent, has been similarly observed in some other studies (Cosme et al. 2022; D'Agostino et al. 2022). Findings that malic acid is dominant have also been identified in studies such as Kafkas et al. (2006) and D'Agostino et al. (2022). The density of malic acid in these fruits plays an important role in terms of the sweetness and acidic properties of the fruit. On the other hand, ascorbic acid content is also found at high levels in the G6 genotype, and this compound contributes to the important health properties of blackberries. Ascorbic acid is a strong antioxidant and can have various positive effects on human health (Gegotek and Skrzydlewska 2022).

**TABLE 6:** Organic acid content (mg 100 g^−1^) in blackberry genotypes naturally grown in Tunceli.

Genotypes	Oxalic acid	Malic acid	Ascorbic acid	Citric acid
G1	0.26b	15.68a	0.89b	0.16a
G2	0.24b	8.48d	0.65c	0.11bc
G3	0.26b	5.86 g	0.46e	0.11bcd
G4	0.23bc	9.53c	0.89b	0.11abc
G5	0.22bc	6.68e	0.59 cd	0.12abc
G6	0.26b	11.00b	1.04a	0.06d
G7	0.33a	5.95 g	0.84b	0.14ab
G8	0.18c	6.14f	0.52de	0.09 cd
G9	0.23bc	8.48d	0.67c	0.13abc

*Note:* Means in columns with the same letter do not differ according to Tukey's test at *p* < 0.05.

### 3.7 Correlation Analysis

**FIGURE 1:**
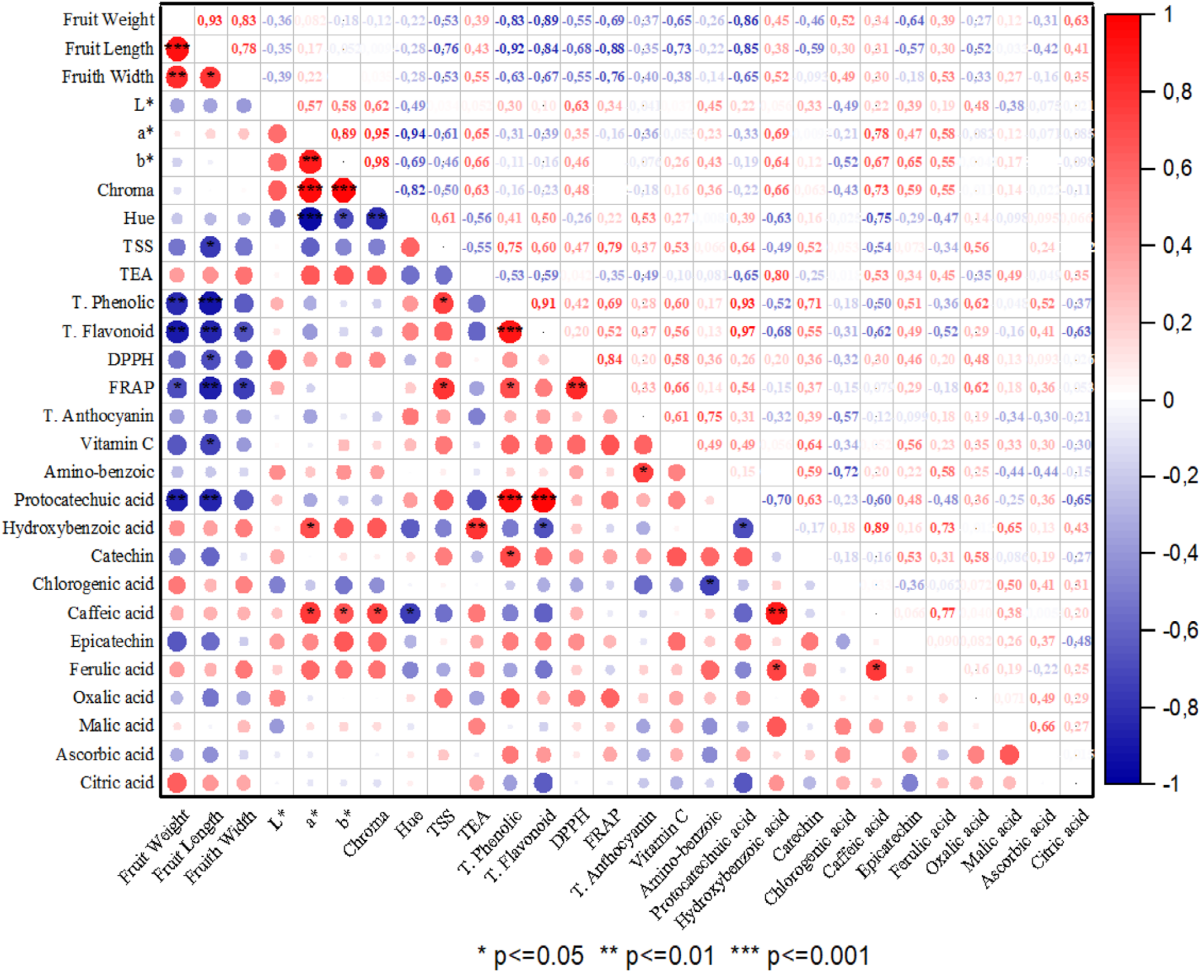
Correlation analysis of traits belonging to blackberry genotypes.

Identifying relationships among the traits studied is crucial for breeding programs. The correlation analysis graph for the traits examined in blackberry genotypes is shown in Figure 1. Positive and negative correlations were observed among the investigated traits. The highest positive correlation was observed between chroma and b* (*r* = 0.98), total flavonoids and protocatechin (*r* = 0.97) and chroma and a* (*r* = 0.95) values, while the highest negative correlation was observed between a* and Hue (*r* = −0.94) and fruit length and total phenolic (*r* = −0.92). In general, fruit dimensions and color values such as *L*, a*, *b*, and Chroma values showed positive correlations among themselves. Total biochemical content values showed negative correlations with fruit dimensions. Fruit weight showed positive correlations with fruit width (*r* = 0.83) and fruit length (*r* = 0.93); fruit length showed positive correlations with fruit width (*r* = 0.78) and a* value and b* value (*r* = 0.89). Among the biochemical contents, there was a positive correlation between total phenolic and protocatechin (*r* = 0.93), total phenolic and total flavonoid (*R* = 0.91), FRAP and DPPH (*r* = 0.84) and caffeic and hydroxybenzoic (*R* = 0.89). Among the color values, hue value showed a negative correlation with all color values. Negative correlations were found between fruit weight and total flavonoids (*r* = −0.89), protocatechin (*r* = −0.86), fruit width (*r* = −0.84) and total phenolics (*r* = −0.83), and between fruit width and FRAP (*r* = −0.88) and protocatechin (*r* = −0.85). Correlations between the examined parameters determine the effect of a trait on other traits. Strong correlations between traits help breeders identify important traits while also speeding up and facilitating breeding efforts (Garazhian et al. 2020). Our findings of correlation among fruit size, TSS, titratable acidity, and biochemical contents were parallel to the findings reported by Gharaghani et al. (2015), Yazdanpour et al. (2018), Garazhian et al. (2020, 2022), and Zhao et al. (2023).

### 3.8 Principal Component Analysis

**FIGURE 2:**
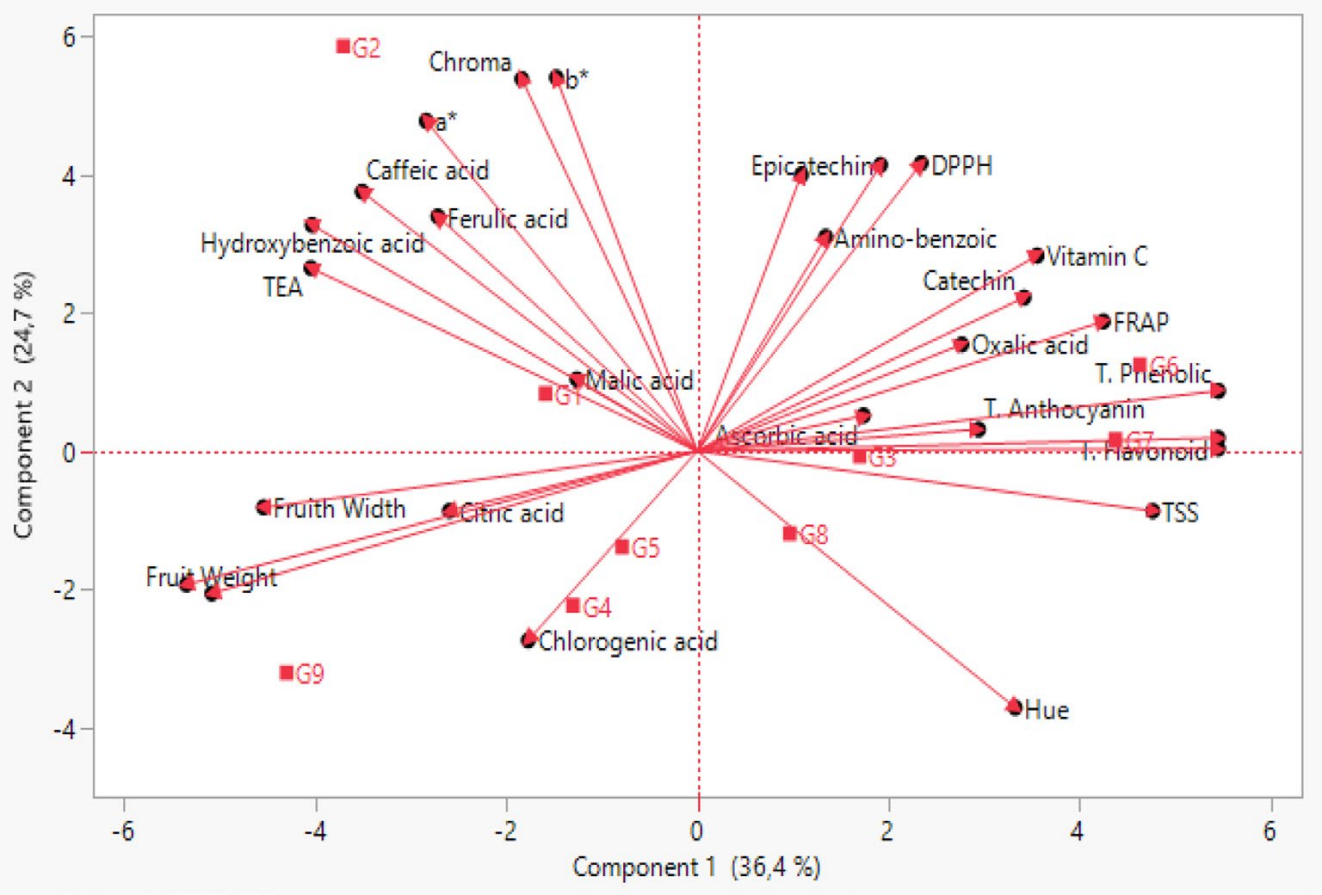
Two‐dimensional principal component analysis graph of the traits of blackberry genotypes.

Principal component analysis (PCA), one of the multivariate statistical methods, is widely used to determine the most important attributes in the data set and to evaluate the germplasm of various plant species. PCA is an effective statistical method to determine the variation in a data set and to reveal strong relationships (Hair et al. 2006). Within the scope of this study, PCA was performed on a total of 28 traits related to the morphological and biochemical properties of blackberry genotypes (Table 7; Figure 2). According to the results of the PCA, six principal components with eigen values > 1 were identified. The first three principal components explained 73.24% of the total variance. This result shows that the traits that are important in the first three principal components have the most diversity among genotypes and have the greatest effect on the differentiation of genotypes (Iezzoni and Pritts 1991). The effects of the traits examined in the first three principal components differed. In the first principal component, which explained 36.37% of the total variance, TSS, TEA, total phenolic, total flavonoid, and protocatechin had positive effects on fruit size, and hydroxybenzoic had negative effects. The Hue value had a negative effect on the second principal component, which explained 24.66% of the total variance, while other color values, DPPH, aminobenzoic, hydroxybenzoic, caffeic, epicatechin, and ferulic, had a positive effect. In the third principal component explaining 3.42% of the total variance, total anthocyanin and aminobenzoic acid had a negative effect, while hydroxybenzoic, chlorgenic, malic acid, and ascorbic acid had a positive effect. In previous studies, the total variance in the first three components was reported as 88.14% by Yazdanpour et al. (2018), 67% by Ochieng et al. (2019), 54.2% by Garazhian et al. (2022) and 63.3% by Zhao et al. (2023). Differences between study results may be due to differences in genotypes and parameters examined in the studies.

**TABLE 7:** Principal component analysis of traits of blackberry genotypes.

Properties	PCA1	%Cont.*	PCA2	%Cont.	PCA3	%Cont.	PCA4	PCA5	PCA6
Fruit weight	**−0.27**	**7.39**	−0.13	1.79	0.02	0.04	0.18	−0.02	0.14
Fruit length	**−0.29**	**8.18**	−0.13	1.57	−0.11	1.12	0.03	0.04	0.07
Fruith width	**−0.24**	**5.90**	−0.05	0.28	0.06	0.38	0.09	0.29	0.32
L*	0.06	0.34	**0.26**	**6.74**	−0.13	1.57	−0.04	−0.42	0.25
a*	−0.15	2.30	**0.31**	**9.63**	−0.03	0.08	−0.15	−0.07	0.10
b*	−0.08	0.62	**0.35**	**12.33**	−0.05	0.28	−0.10	0.05	−0.07
Chroma	−0.10	0.97	**0.35**	**12.24**	−0.04	0.19	−0.13	−0.03	−0.03
Hue	0.18	3.17	**−0.24**	**5.82**	−0.01	0.00	0.19	0.17	−0.14
TSS	**0.26**	**6.51**	−0.06	0.32	0.14	2.08	0.21	−0.09	−0.03
TEA	**−0.22**	**4.68**	0.17	2.96	0.11	1.24	−0.08	0.13	−0.13
T. phenolics	**0.29**	**8.52**	0.06	0.32	0.11	1.30	−0.04	0.01	0.19
T. flavonoids	**0.29**	**8.51**	0.00	0.00	0.00	0.00	−0.17	0.15	0.05
DPPH	0.13	1.57	**0.27**	**7.33**	0.08	0.69	0.13	−0.26	−0.20
FRAP	0.23	**5.17**	0.12	1.48	0.18	3.20	0.15	−0.23	−0.25
T. anthocyanin	0.16	2.48	0.02	0.04	**−0.27**	**7.43**	0.34	0.20	−0.21
Vitamin C	0.19	3.62	0.18	3.38	0.09	0.80	0.19	0.33	−0.21
Amino‐benzoic	0.07	0.52	**0.20**	**4.06**	**−0.34**	**11.56**	0.29	0.14	0.09
Protocatechuic acid	**0.29**	**8.51**	0.01	0.02	0.00	0.00	−0.16	0.07	0.18
Hydroxybenzoic acid	**−0.22**	**4.66**	**0.21**	**4.52**	**0.20**	**4.08**	0.13	0.07	−0.10
Catechin	0.18	3.35	0.14	2.08	0.00	0.00	0.18	0.24	0.46
Chlorogenic acid	−0.09	0.90	−0.18	3.17	**0.38**	**14.20**	0.03	−0.02	0.24
Caffeic acid	−0.19	3.52	**0.24**	**5.94**	0.07	0.45	0.13	−0.03	−0.09
Epicatechin	0.10	1.05	**0.27**	**7.24**	0.09	0.88	−0.26	0.24	0.08
Ferulic acid	−0.15	2.11	**0.22**	**4.87**	−0.05	0.23	0.34	0.19	0.18
Oxalic acid	0.15	2.19	0.10	1.01	0.19	3.53	0.30	−0.25	0.33
Malic acid	−0.07	0.46	0.07	0.46	**0.47**	**21.86**	0.04	0.23	−0.22
Ascorbic acid	0.09	0.87	0.03	0.11	**0.45**	**19.92**	−0.11	0.07	0.11
Citric acid	−0.14	1.93	−0.06	0.32	0.17	2.87	0.36	−0.27	−0.02
Eigenvalue	10.18		6.90		3.42		2.63	1.88	1.43
Variability (%)	36.37		24.66		12.21		9.41	6.71	5.12
Cumulative %	36.37		61.03		73.24		82.65	89.36	94.48

*Note:* * Cont: Contribution. Bold values are statistically significant.

### 3.9 Heatmap Hierarchical Clustering Analysis

**FIGURE 3:**
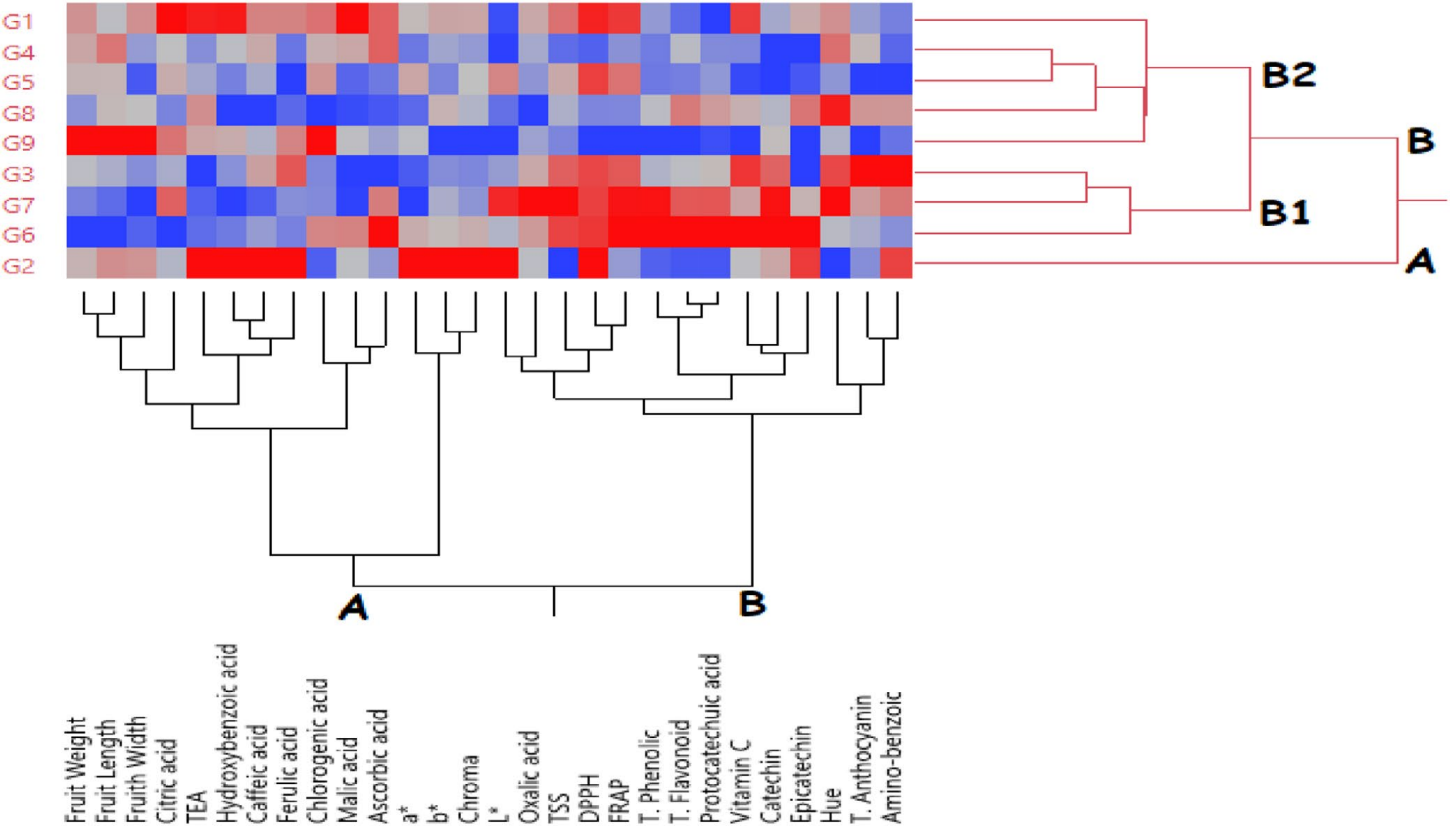
Heatmap hierarchical clustering analysis of the examined traits of blackberry genotypes.

Heatmap hierarchical clustering analysis was performed to classify the examined blackberry genotypes according to their morphological, biochemical, individual phenolic, and organic acid content characteristics (Figure 3). In the heatmap analysis, the color intensity changing from blue to red indicates the high trait values of the genotypes. As a result of the heatmap hierarchical clustering analysis, the genotypes were divided into two main groups (Figure 3). The G2 genotype formed group A separately from the other genotypes. The other genotypes formed group B. Group B was divided into subgroups within itself. The G2 genotype had high values in TEA, hydroxybenzoic, caffeic, ferulic, a*, b*, chroma, L*, DPPH, epicatechin, and aminobenzoic traits. The B1 group exhibited relatively higher values in terms of aminobenzoic, Hue, epicatechin, catechin, vitamin C, protocatechin, total flavonoid, total phenolic, FRAP, DPPH, TSS, oxalic acid, and L* values, while the B2 group exhibited relatively higher values. Heatmap hierarchical clustering analysis has been widely used to cluster genotypes or varieties according to their characteristics in fruit species such as blackberry (Tas 2023), strawberry (Elikara et al. 2024), oleaster (Say et al. 2024) and walnut (Kömür et al. 2024).

## 4 Conclusion

In this study, blackberry genotypes naturally grown in Tunceli were examined and superior characteristics were determined. The results showed that there were significant differences among the genotypes in terms of fruit weight, length, width, color values, TSS, titratable acidity, vitamin C, antioxidant activity, organic acids, and individual phenolic compounds. In particular, the G9 genotype had the largest fruits, while the G6 genotype stood out with its high phenolic, flavonoid, and anthocyanin content, and the G2 genotype attracted attention with its high color values and acidity content.

The research findings revealed that the levels of phenolic compounds in different blackberry genotypes showed great variability. While the G1, G2, and G6 genotypes were distinguished by their high phenolic compound content, these levels were found to be low in the G4 and G5 genotypes. In addition, the G7 genotype had the highest level of oxalic acid, while G8 had the lowest level. In terms of other acids, G1 was determined to be the richest genotype in malic acid, while G6 showed the highest value in ascorbic acid. In conclusion, this study shows that the G9, G6, G2, and G1 genotypes stand out with their superior characteristics and that these genotypes have potential in terms of agricultural production.

## Author Contributions

**Hande Dogan:** conceptualization (equal), investigation (equal), methodology (equal), resources (equal), validation (equal), writing – original draft (equal). **Erdal Aglar:** investigation (equal), resources (equal), validation (equal), writing – original draft (equal), writing – review and editing (equal). **Burhan Ozturk:** methodology (equal), supervision (equal), writing – original draft (equal), writing – review and editing (equal). **Onur Tekin:** data curation (equal), formal analysis (equal), methodology (equal), resources (equal), software (equal). **Davut Alan:** data curation (equal), formal analysis (equal), methodology (equal). **Ahmet Sumbul:** conceptualization (equal), data curation (equal), formal analysis (equal), validation (equal).

## Ethics Statement

The plant material of the study consisted of blackberry genotypes naturally grown in Karsılar village of the central district of Tunceli. Moreover, we have obtained appropriate permission from the responsible committee to collect the plant material from the orchard.

## Consent

The authors have nothing to report.

## Data Availability

All data generated or analyzed during this study are included in this published article.
